# Decline in Health-Related Quality of Life reported by more than half of those waiting for joint replacement surgery: a prospective cohort study

**DOI:** 10.1186/1471-2474-12-108

**Published:** 2011-05-23

**Authors:** Ilana N Ackerman, Kim L Bennell, Richard H Osborne

**Affiliations:** 1 Centre for Clinical Epidemiology, Biostatistics and Health Services Research, Department of Medicine (Royal Melbourne Hospital), The University of Melbourne, Parkville, Australia; 2 Centre for Health, Exercise and Sports Medicine, School of Physiotherapy, The University of Melbourne, Parkville, Australia; 3 Public Health Innovation, Population Health Strategic Research Centre, Deakin University, Burwood, Australia

## Abstract

**Background:**

In many healthcare systems, people with severe joint disease wait months to years for joint replacement surgery. There are little empirical data on the health consequences of this delay and it is unclear whether people with substantial morbidity at entry to the waiting list continue to deteriorate further while awaiting surgery. This study investigated changes in Health-Related Quality of Life (HRQoL), health status and psychological distress among people waiting for total hip (THR) and knee replacement (TKR) surgery at a major metropolitan Australian public hospital.

**Methods:**

134 patients completed questionnaires including the Assessment of Quality of Life (AQoL) instrument, Western Ontario and McMaster Universities Osteoarthritis Index (WOMAC) and Kessler Psychological Distress Scale after entering an orthopaedic waiting list (baseline) and before surgery (preadmission). To quantify potential decline in wellbeing, we calculated the proportion of people experiencing clinically important deterioration using published guidelines and compared HRQoL and psychological distress outcomes with population norms.

**Results:**

Most participants (69%) waited ≥6 months for surgery (median 286 days, IQR 169-375 days). Despite poor physical and psychological wellbeing at baseline, there was an overall deterioration in HRQoL during the waiting period (mean AQoL change -0.04, 95%CI -0.08 to -0.01), with 53% of participants experiencing decline in HRQoL (≥0.04 AQoL units). HRQoL prior to surgery remained substantially lower than Australian population norms (mean sample AQoL 0.37, 95%CI 0.33 to 0.42 vs mean population AQoL 0.83, 95%CI 0.82 to 0.84). Twenty-five per cent of participants showed decline in health status (≥9.6 WOMAC units) over the waiting period and prevalence of high psychological distress remained high at preadmission (RR 3.5, 95%CI 2.8 to 4.5). Most participants considered their pain (84%), fatigue (76%), quality of life (73%) and confidence in managing their health (55%) had worsened while waiting for surgery.

**Conclusions:**

Despite substantial initial morbidity, over half of the participants awaiting joint replacement experienced deterioration in HRQoL during the waiting period. These data provide much-needed evidence to guide health professionals and policymakers in the design of care pathways and resource allocation for people who require joint replacement surgery.

## Background

As in many developed countries, demand for joint replacement surgery in Australia has grown rapidly. The number of joint replacements performed has doubled over the past 12 years [1] and further increases are expected in light of the ageing population and increasing risk factors for osteoarthritis [2]. A mismatch between demand for surgery and service provision has resulted in lengthy waiting lists for primary total hip (THR) and knee replacement (TKR) in the Australian public hospital system [3], representing a major public health and political issue unlikely to abate in the near future. Similar situations exist in other countries including Canada [4] and the United Kingdom (UK) [5].

Our earlier research showed that people entering an Australian orthopaedic waiting list for joint replacement had severely compromised Health-Related Quality of Life (HRQoL) and higher levels of psychological distress compared with the general population [6]. More recently, poor HRQoL has also been reported in a cross-sectional study of Canadian patients entering a waiting list for TKR [7]. However, it is unclear whether people with substantial morbidity at entry to the waiting list continue to deteriorate further while awaiting surgery. Further decline could potentially result from increasing joint disease severity over time and the associated difficulty in maintaining activity levels, employment and family and community roles. Although a range of studies have prospectively evaluated changes in the health status of people waiting for joint replacement [8-13], results have been conflicting and average waiting times relatively short (range 2-6 months). Hoogeboom et al [14] were unable to perform a meta-analysis due to the heterogeneity of studies in this field but their published 'qualitative data analysis' concluded that neither pain (for people awaiting THR or TKR) nor function (for people awaiting THR) deteriorated when waiting less than 6 months for surgery. The effect of waiting longer than 6 months could not be determined. Indeed, little research has been conducted into the impact of longer waiting times for joint replacement on patient wellbeing. A Swedish study found that people waiting, on average, 8 months for THR experienced significant deterioration in HRQoL although changes in pain and function over the waiting period are unclear as these analyses were not specifically reported [15]. Research from the UK involving people waiting for THR or TKR revealed small fluctuations in pain and physical function over the waiting period although less than 50% of participants were available for follow-up after 6 months of waiting and only 23% were available at the 9-month time point [16]. Most recently, Desmeules et al [13] reported deterioration in pain and physical function among people waiting more than 9 months for TKR in Canada; however, as data were only reported for 3 of the 8 Medical Outcomes Study Short Form Health Survey (SF-36) dimensions it is difficult to evaluate change in overall HRQoL.

As pre-operative wellbeing is an important predictor of joint replacement outcomes [9,17,18], knowledge of changes in health status represents valuable information for patients, clinicians and health policy makers and offers potential avenues for future interventions. To inform clinical practice and health policy, this study investigated changes in key indicators of wellbeing (HRQoL, health status, psychological distress and self-perceived change) among people awaiting THR or TKR.

## Methods

### Participants

This study comprised a cohort of patients awaiting joint replacement at the Royal Melbourne Hospital, Victoria, Australia (a large metropolitan public teaching hospital). Patients entered the orthopaedic waiting list following referral from a general practitioner and consultation with an orthopaedic surgeon. The study recruitment procedures have been described previously [6]; all patients on the waiting list for unilateral primary THR or TKR as of December 2002 were contacted about the study. Furthermore, from December 2002 to June 2005, all patients sequentially added to the waiting list were contacted within one week. No sample size target was specified *a priori*. Patients were eligible to participate if aged over 18 years, awaiting unilateral primary THR or TKR and fluent in English. Exclusion criteria included overt evidence of cognitive dysfunction, surgery scheduled within 30 days or severe medical illness precluding participation. The study was approved by the Melbourne Health Human Research Ethics Committee. All data collection was undertaken from 2002 to 2005.

**Figure 1:**
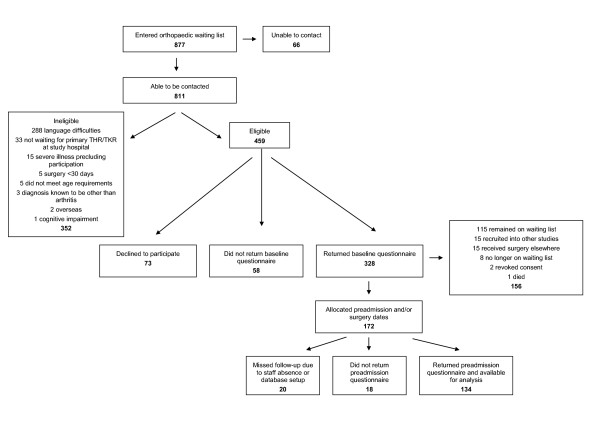
**Participant numbers**.

Eligible patients who provided verbal consent were mailed a consent form and baseline questionnaire (baseline assessment). A preadmission questionnaire was mailed once a preadmission clinic appointment had been scheduled, generally 2-6 weeks before surgery (preadmission assessment). Missing data were followed-up by telephone or mail, where possible. Baseline data were collected from 328 participants; however, similar to previous research in this field [12], a considerable number of participants were not available for follow-up. This was predominantly due to participants having not been scheduled for surgery by the end of the 2.5 year data collection period (Figure 1). This paper reports 134 participants who completed baseline and preadmission assessments.

### Questionnaires

HRQoL was assessed using the 12-item generic Assessment of Quality of Life (AQoL) instrument [19]. The AQoL has good psychometric properties and is more responsive than other widely-used generic scales, including the SF-36 [20,21]. It produces a utility value ranging from -0.04 (worst HRQoL) to 1.00 (full HRQoL).

The Western Ontario and McMaster Universities Osteoarthritis (WOMAC) Index is an osteoarthritis-specific health status instrument; its validity, reliability and responsiveness have been extensively demonstrated [22]. The WOMAC consists of 24 questions and produces a total score which was transformed to a 0 (best health state) to 100 (worst health state) scale.

The Kessler Psychological Distress Scale (K10) is used in Australian population health surveys and the World Health Organisation World Mental Health Survey [23]. The K10 consists of 10 questions and produces a score ranging from 10 (lowest psychological distress) to 50 (highest psychological distress). High K10 scores are strong predictors of affective disorders such as depression and anxiety [24].

Demographic information including education, marital status and employment status and past medical history were collected at baseline. Additional information extracted from patient records included date of birth, date of entry to the waiting list, preadmission clinic and surgery dates and surgery type. At preadmission, participants were also asked to rate their self-perceived change (if any) in pain, fatigue, overall quality of life, overall health and confidence in managing their health since entering the waiting list. Self-perceived change was assessed on a 7-point scale ranging from 'a great deal worse' to 'a great deal better'.

### Statistical analyses

Analyses were undertaken using Statistical Package for the Social Sciences (SPSS) Version 14.0. Questionnaire scores were calculated according to published algorithms [24-26]. Missing values were imputed using the mean values of the remaining items, provided that a sufficient proportion of remaining items were available, as specified by the scoring guidelines for each measure. Mann Whitney, chi square and unpaired t-tests were used to compare demographic and baseline data from participants who completed baseline and preadmission questionnaires with those who provided baseline data only. Changes in HRQoL, health status and psychological distress from baseline to preadmission were examined using paired t-tests. Effect sizes were used to estimate the magnitude of overall change (calculated by dividing the change score for each measure by the baseline standard deviation) [27]. Self-perceived change data were analysed descriptively.

The proportion of people who experienced clinically important change in HRQoL and health status was calculated according to published data for the AQoL and WOMAC instruments. Clinically important deterioration in HRQoL was defined as a decrease of ≥0.04 AQoL units and improvement as an increase of ≥0.04 AQoL units [28]. Clinically important deterioration in health status was defined as an increase of ≥9.6 WOMAC units and improvement as a decrease of ≥8.2 WOMAC units [29]. The relationship between baseline status and category of clinically important change for the AQoL and WOMAC measures was investigated using analysis of covariance, with adjustment for age and gender. Separate analyses for THR and TKR were not undertaken due to the limited sample size. The potential contribution of regression to the mean (RTM) was evaluated by repeating the paired t-test analyses after excluding extreme baseline scores (the top 5% and the bottom 5% of baseline scores for each measure). RTM is a statistical phenomenon which may occur with repeated measurements, where extreme measures obtained at the first time point are likely to be closer to the sample mean on subsequent testing due to random error [30]. This is particularly relevant for the present study which involves participants with very poor wellbeing at baseline (as only those with severe joint disease enter the orthopaedic waiting list). Although a definitive test is not available, the exclusion of extreme scores was used as scores at the upper and lower limits of a scale are theoretically most susceptible to RTM [30].

Baseline and preadmission K10 scores were categorised into levels of psychological distress according to published definitions [31] and compared with data from the Victorian Population Health Survey [32]. Relative risk (RR) for presence of high/very high psychological distress (K10 ≥22) compared with population levels was calculated using Confidence Interval Analysis software (Version 2.0.0) [33].

## Results

### Comparison of demographic and baseline data

Table 1 shows that participants who completed baseline and preadmission questionnaires (*n *= 134) were similar to those who completed a baseline questionnaire only (*n *= 194) across a range of demographic and baseline characteristics. This suggests that the sample can be considered broadly representative of our overall cohort at baseline.

**Table 1:** Comparison of demographic and baseline characteristics

Characteristic	Baseline and preadmission data (*n *= 134*)	Baseline data only (*n *= 194*)	p
Age in years: median (IQR)	67 (61-75)	68 (61-75)	0.87^†^
Female: number (%)	79 (59)	108 (56)	0.56^‡^
Waiting for TKR: number (%)	69 (52)	110 (57)	0.35^‡^
Married or living with partner: number (%)	85 (64)	114 (59)	0.35^‡^
Highest educational level completed: number (%)			0.50^‡^
Primary school or less	41 (31)	52 (27)	
Years 7-10	51 (39)	77 (40)	
Years 11-12	25 (19)	31 (16)	
Trade/technical/university	15 (11)	32 (17)	
Employment status: number (%)			0.25^‡^
Retired/receiving aged pension	92 (70)	141 (73)	
Not in paid work	28 (21)	29 (15)	
Paid work	11 (8)	23 (12)	
Income: number (%)			0.52^‡^
<$AUD 10,000	17 (18)	37 (24)	
$AUD 10,000 - 19,999	46 (49)	68 (44)	
$AUD 20,000 and over	31 (33)	48 (31)	
Number of co-morbidities: median (IQR)	1 (0-2)	1 (0-2)	0.36^†^
Baseline AQoL score: mean (SD)	0.42 (0.23)	0.39 (0.26)	0.35^#^
Baseline WOMAC score: mean (SD)	59.8 (18.4)	58.8 (18.5)	0.64^#^
Baseline K10 score: median (IQR)	18.0 (14.0-25.0)	19.0 (14.0-27.0)	0.52^†^

*Numbers for each characteristic may not total 134 or 194, respectively, due to missing responses

^†^Mann Whitney test

^‡^Chi square test

^#^Unpaired t-test

### Demographics and waiting times

Demographic data for the study sample are presented in Table 1. The median (IQR) time from entry to the waiting list to surgery was 286 (169-375) days (range 43-1069 days). Twenty-five per cent of participants waited less than 6 months for surgery (*n *= 33), 43% (*n *= 57) waited 6-12 months and 26% (*n *= 35) waited more than 12 months. Waiting time data were not available for 9 participants (7%). The median (IQR) time between baseline and preadmission questionnaires was 165 (84-268) days, with preadmission questionnaires completed, on average, 1 month before surgery (median 30 days, IQR 18-49 days).

Seventy-eight per cent of participants (*n *= 104) had self-reported osteoarthritis and 18% (*n *= 24) reported rheumatoid arthritis. Other concurrent musculoskeletal conditions included back pain (*n *= 59, 44%), osteoporosis (*n *= 18, 13%), gout (*n *= 9, 7%) and fibromyalgia (*n *= 1, <1%). Twenty per cent (*n *= 27) had received a previous joint replacement. Using a checklist of 6 common conditions, 40% of participants (*n *= 53) had hypertension, 13% (*n *= 18) had diabetes, 13% (*n *= 17) had coronary artery disease, 12% (*n *= 16) had asthma, 11% (*n *= 15) had anxiety or depression and 4% (*n *= 5) had cancer.

### Changes in wellbeing from baseline to preadmission

Table 2 shows that there was a clinically important decline in HRQoL from baseline to preadmission. Although the effect size for deterioration in HRQoL was small, from a clinical perspective, this represents 22% of the magnitude of change in HRQoL 3 months after THR or TKR (effect size 0.86) [34]. Preadmission HRQoL for the combined sample (THR and TKR) was extremely poor when compared with Australian population norms (mean preadmission AQoL score 0.37, 95%CI 0.33 to 0.42 versus mean population AQoL score 0.83, 95%CI 0.82 to 0.84) [28]. Separate analyses by operation type (THR or TKR) revealed that participants awaiting THR experienced a decline in HRQoL (Table 2). The magnitude of this effect size represents almost one-third of the change in HRQoL from joint replacement [34]. On average, participants awaiting TKR experienced little change in HRQoL (Table 2).

**Table 2:** Change in Health-Related Quality of Life, health status and psychological distress

		Baseline		Preadmission		Change		**Effect size**^**†**^	p**
**Construct**^**‡**^	n*	Mean	SD	Mean	SD	**Mean**^**#**^	95% CI		
Health-Related Quality of Life (AQoL)									
Overall sample	127	0.42	0.22	0.37	0.25	-0.04	-0.08 to -0.01	-0.19	0.02
Total hip replacement	63	0.42	0.23	0.36	0.24	-0.06	-0.11 to -0.02	-0.27	0.01
Total knee replacement	64	0.41	0.22	0.39	0.26	-0.02	-0.08 to 0.03	-0.10	0.39
Health status (WOMAC)									
Overall sample	119	59.7	18.6	61.3	18.0	1.5	-1.1 to 4.2	-0.08	0.26
Total hip replacement	61	60.5	20.5	62.1	18.6	1.7	-1.9 to 5.2	-0.08	0.35
Total knee replacement	58	59.0	16.5	60.3	17.4	1.4	-2.7 to 5.4	-0.08	0.50
Psychological distress (K10)									
Overall sample	132	20.2	7.7	20.7	8.0	0.5	-0.4 to 1.4	-0.07	0.25
Total hip replacement	65	19.7	7.9	20.2	8.0	0.5	-0.9 to 1.9	-0.06	0.50
Total knee replacement	67	20.7	7.6	21.3	8.0	0.5	-0.6 to 1.7	-0.07	0.33

^‡^AQoL range: -0.04 (worst HRQoL) to 1.00 (full HRQoL); WOMAC range: 0 (best health state) to 100 (worst health state); K10 range: 10 (lowest psychological distress) to 50 (highest psychological distress)

*Number of participants with complete baseline and preadmission data for each measure; does not total 134 due to missing data

^#^Negative change score indicates deterioration for AQoL; positive change score indicates deterioration for WOMAC and K10

^†^Negative effect size indicates deterioration

**Paired t-test

While the combined sample demonstrated poor health status at baseline, there was little overall change in WOMAC score over the waiting period. Similarly, there was minimal overall change in psychological distress from baseline to preadmission. Separate analysis of THR and TKR data produced similar results (Table 2).

Missing item responses were infrequent for the AQoL (2% of items at baseline; 1% of items at preadmission), WOMAC (3% at baseline; 4% at preadmission) and K10 (0% at baseline; <1% at preadmission) instruments.

### Proportion of participants who experienced clinically important change

Table 3 shows the variability in wellbeing over the waiting period. A substantial proportion of the combined sample (53%) experienced a clinically important decline in HRQoL while awaiting surgery (56% for THR; 50% for TKR). Conversely, 29% experienced clinically important improvement in HRQoL (25% for THR; 33% for TKR) while 18% had no change (19% for THR; 17% for TKR).

**Table 3:** Proportion of participants reporting clinically important change

Construct	Category		
	Decline* *n *(%)	No change *n *(%)	**Improvement**^**† **^***n *(%)**
Health-Related Quality of Life (AQoL)			
Overall sample	67 (53)	23 (18)	37 (29)
Total hip replacement	35 (56)	12 (19)	16 (25)
Total knee replacement	32 (50)	11 (17)	21 (33)
Health status (WOMAC Index)			
Overall sample	30 (25)	61 (51)	28 (24)
Total hip replacement	16 (26)	31 (51)	14 (23)
Total knee replacement	14 (24)	30 (52)	14 (24)

*Decrease of ≥0.04 units for AQoL instrument; increase of ≥9.6 units for WOMAC Index

^**†**^Increase of ≥0.04 units for AQoL; decrease of ≥8.2 units for WOMAC Index

Twenty-five per cent of participants had a clinically important deterioration in health status (26% for THR; 24% for TKR), 24% experienced clinically important improvement (23% for THR; 24% for TKR) and 51% had no change in health status (51% for THR; 52% for TKR).

### Sensitivity analysis

After adjusting for age and gender, participants who experienced a clinically important decline in HRQoL had the highest HRQoL at baseline, while those who had a clinically important improvement in HRQoL reported the lowest HRQoL at baseline (mean (SD) baseline AQoL score 0.47 (0.19) versus 0.35 (0.22); F = 3.54, p = 0.03). As a similar pattern was seen for health status (mean (SD) baseline WOMAC score 46.9 (16.5) versus 70.3 (19.5); F = 14.32, p < 0.01), it is possible that these findings may be partly related to RTM. After excluding extreme baseline scores and repeating the paired t-test analyses, the magnitude of baseline to preadmission change was found to be the same or larger than for the original analyses (effect size -0.26 for AQoL, -0.14 for WOMAC and -0.07 for K10). This suggests that the effect of RTM was likely to be minimal and that it may have (to a small degree) masked the true magnitude of change during the waiting period. Additionally, as the sample reported, on average, very poor HRQoL at baseline, the net effect of RTM should have produced an overall trend towards improvement in HRQoL; in contrast, HRQoL deteriorated significantly from baseline to preadmission.

### Risk of psychological distress

For the combined sample, high or very high psychological distress remained more prevalent compared with the general population at preadmission (RR 3.5, 95%CI 2.8 to 4.5). Relative risk (95%CI) was 3.4 (2.4 to 4.8) for participants awaiting THR and 3.7 (2.7 to 5.1) for participants awaiting TKR.

### Self-perceived change in wellbeing

Table 4 shows that most participants perceived that their pain, fatigue, overall quality of life, overall health and confidence in managing their health had worsened since entering the waiting list. At the extreme end of the scale, over one-third (36%) considered their pain was 'a great deal worse'; while 17% reported their overall quality of life was 'a great deal worse'. Separate analysis by operation type (THR or TKR) produced similar results, although a greater proportion of participants awaiting THR considered their overall quality of life was 'a great deal worse' (24% versus 10% for TKR).

**Table 4:** Self-perceived change in wellbeing

Construct	Worse*		No change		**Better**^**#**^	
	*n*	**%**^**†**^	*n*	**%**^**†**^	*n*	**%**^**†**^
Pain						
Overall sample	112	85	15	11	5	4
Total hip replacement	59	92	5	8	0	0
Total knee replacement	53	78	10	15	5	7
Fatigue						
Overall sample	100	76	27	21	5	4
Total hip replacement	49	77	14	22	1	2
Total knee replacement	51	75	13	19	4	6
Overall quality of life						
Overall sample	96	73	30	23	6	5
Total hip replacement	48	76	14	22	1	2
Total knee replacement	48	70	16	23	5	7
Overall health						
Overall sample	72	55	49	37	10	8
Total hip replacement	34	54	26	41	3	5
Total knee replacement	38	56	23	34	7	10
Confidence in managing one's health						
Overall sample	74	56	46	35	13	10
Total hip replacement	37	58	20	31	7	11
Total knee replacement	37	54	26	38	6	9

*Combines 'A great deal worse', 'Moderately worse' and 'A little worse' categories

^#^Combines 'A little better', 'Moderately better' and 'A great deal better' categories

^†^May not total 100% due to rounding

## Discussion

This study demonstrates that despite substantial initial morbidity, 53% of people awaiting joint replacement experienced deterioration in HRQoL over the waiting period. Life quality before surgery was markedly lower than Australian population norms [28] and the prevalence of high psychological distress remained higher than for the general population. In general, participants perceived their pain, fatigue, quality of life, overall health and confidence in managing their health had worsened while waiting. These findings are concerning given that pre-operative wellbeing is a strong predictor of outcome after joint replacement surgery [18,35,36].

It is unclear why people awaiting THR reported, on average, greater deterioration in HRQoL than people waiting for TKR. This finding was also supported by the self-perceived change data. Possible contributing factors such as age and number of co-morbidities were similar for both groups (data not shown) and people awaiting TKR had a longer median waiting time (309 days for TKR versus 243 days for THR). Deterioration in HRQoL has also been reported in people awaiting THR in the Netherlands [9] and Sweden [15] using a different HRQoL utility measure, the EQ-5D. Other studies have investigated HRQoL in people awaiting joint replacement but cannot be directly compared with our findings. In one, utility scores for the EQ-5D were not reported and as deterioration was observed in only one of five EQ-5D dimensions [10], it is unlikely that this would result in HRQoL decline. Hirvonen et al [11] did not observe a change in HRQoL (assessed using the 15D utility instrument) among people awaiting THR or TKR in Finland, although most participants (71%) waited 3 months or less, which is considerably shorter than in Australia, Canada and the United Kingdom. Using the SF-36 instrument, McHugh et al [16] found little change in HRQoL among people waiting for THR and TKR, although these data were not analysed separately. Most recently, Desmeules et al [13] also assessed HRQoL in people waiting for TKR but only reported change data for 3 of the SF-36 dimensions.

The finding of significant deterioration in HRQoL without a corresponding change in health status or psychological distress scores does appear to be counter-intuitive. However, for both the WOMAC and K10 measures, there was an overall trend towards deterioration from baseline to preadmission although the magnitude of change was small and unlikely to be clinically relevant (effect sizes -0.08 and -0.07, respectively). As a generic measure of HRQoL, the AQoL instrument provides different information to that obtained from the other instruments and this may partly explain our findings. For example, the AQoL incorporates a number of constructs relating to physical functioning that are not assessed by the WOMAC but that may be impacted by severe joint disease, such as the level of assistance required for personal care and household tasks. Similarly, it also covers areas relating to psychosocial wellbeing which are not included in the K10, such as relationships with others (as affected by health), social isolation, the capacity to undertake one's role within the family and the ability to sleep. Although the impact of longer waiting times has been unclear [14], several studies have reported deterioration in WOMAC pain and/or physical function subscale scores among people waiting for joint replacement. While these changes have been statistically significant, the mean changes in WOMAC scores have tended to be small [9,10,13,16]. In contrast, an earlier Canadian study found no change in WOMAC scores over the waiting period [8]. Interestingly, the present cohort had worse baseline WOMAC pain and physical function subscale scores (data not shown) than reported for other cohorts [8,10,12,13] indicating greater initial morbidity. Previous studies investigating psychological wellbeing have produced conflicting results and are not directly comparable with the present study due to the different measures used. Kelly et al [8] reported improvements in SF-36 mental health and role emotional dimension scores for people awaiting TKR, while those awaiting THR did not change. In contrast, another study involving people waiting for THR found a small decline in SF-36 mental health scores [9]. More recently, McHugh et al [16] reported minimal deterioration in SF-36 mental health scores after 6 months of waiting for joint replacement, while role emotional scores improved.

It is possible that health status and psychological distress fluctuated over the waiting period, similar to the fluctuations in pain and physical function previously reported for this patient group [16]; however, our methodology allowed us to evaluate overall change only. Ideally, regular assessments (eg. 6 monthly) could be used to determine *rate *of change. Also, the present study was only conducted at one public hospital, although baseline HRQoL was comparable to that of people awaiting joint replacement at other large public hospitals in the state of Victoria (range of mean AQoL scores from five Victorian public hospitals = 0.33 to 0.39, Osborne et al, unpublished data). Another limitation reflecting the 'real world setting' of this study is the considerable number of participants who were not scheduled for surgery by the end of the 2.5 year data collection period (at the time of this study there was no standardised process at the study hospital for determining surgical priority and/or exit from the waiting list). However, comparison of demographic and baseline data indicated that the study sample was representative of our overall cohort.

Our use of transition questions to explore self-perceived change across a range of constructs was a novel approach in this field; however, these results should be interpreted cautiously as they rely on participant recall [37] and may be subject to bias including response shift [38]. It is unlikely; however, that participants reported worsening to receive earlier surgery as preadmission questionnaires were only administered once surgery was imminent. Floor or ceiling effects are also unlikely to have impacted on the findings as few participants reported minimum or maximum possible scores for any of the outcome measures (data not shown). It should be acknowledged that the present data are self-reported and further research involving performance-based measures would provide additional information to improve our understanding of changes in physical function while waiting for surgery. In this study, participants were asked to report their use of medications at each assessment; however, given the variability in the type (prescribed, over-the-counter and complementary) and dosages of medications reported, these data were not included in the analyses. Additionally, participants were asked to report the number of visits to a general practitioner, orthopaedic surgeon, rheumatologist or physiotherapist at baseline and preadmission; on average, there were few visits to any of these health professionals at either point (data not shown). However, it should be acknowledged that some participants did use medications (such as paracetamol and non-steroidal anti-inflammatory drugs) and other conservative therapies and that the negative effects of waiting for surgery might be worse in the absence of any therapy. Finally, the results are also likely to represent a conservative estimate of deterioration given that only the distal portion of the entire waiting period was assessed (for example, time spent waiting for an initial consultation with an orthopaedic surgeon was not included) and preadmission clinic dates were used to approximate the end of the waiting period.

Despite these limitations, this study has informed aspects of local health policy and patient care. In the state of Victoria, Australia, the Victorian Government has implemented a program to improve the management and prioritisation of people referred to orthopaedic waiting lists [39]. This program involved the development of a new surgical prioritisation tool (the Multi-Attribute Prioritisation Tool) [40] and service delivery model (the OA Hip and Knee Service) which are now being introduced into major public hospitals in several Australian states. As part of the new system, musculoskeletal co-ordinators periodically assess people awaiting joint replacement to identify deterioration (or improvement) and initiate appropriate services or fast-track surgery as required. Routine, effective prioritisation together with clinical assessment also ensures more timely and equitable access to surgery for those with the greatest need.

## Conclusions

This research has revealed that more than half of those waiting for joint replacement experienced substantial deterioration in HRQoL. While there was little overall change in health status and psychological distress over the waiting period, most people perceived that other aspects of their wellbeing including pain, fatigue and confidence in managing their health had worsened while waiting. By highlighting the severe morbidity of this patient group and the need for periodic monitoring, this research has provided much-needed evidence to guide health professionals and policymakers in the development of care pathways and resource allocation.

## Competing interests

The authors declare that they have no competing interests.

## Authors' contributions

All authors contributed to the study design, interpretation of the results and drafting of the manuscript. Data collection and statistical analysis were undertaken by INA. All authors approved the final manuscript.

## Pre-publication history

The pre-publication history for this paper can be accessed here:

http://www.biomedcentral.com/1471-2474/12/108/prepub
